# The intestinal microbial community dissimilarity in hepatitis B virus-related liver cirrhosis patients with and without at alcohol consumption

**DOI:** 10.1186/s13099-019-0337-2

**Published:** 2019-11-26

**Authors:** Yong-Dong Deng, Xue-Bin Peng, Rong-Rong Zhao, Chao-Qun Ma, Jian-ning Li, Li-Qiong Yao

**Affiliations:** 1 Department of Infection, First Hospital of Lanzhou University, #1 Donggang West Road, Lanzhou, 730000 Gansu China; 2 First Clinical Medicine of Lanzhou University, Medical College of Lanzhou University, #199 Donggang West Road, Lanzhou, 730000 Gansu China; 3 Department of Laboratory, First Hospital of Lanzhou University, #1 Donggang West Road, Lanzhou, 730000 Gansu China

**Keywords:** Hepatitis B virus-related liver cirrhosis, Intestinal microbiota, 16S rRNA gene sequencing, Child–Pugh class

## Abstract

**Background:**

Chronic hepatitis B virus (HBV) infection-reduced liver functions are associated with intestinal microbial community dissimilarity. This study aimed to investigate the microbial community dissimilarity in patients with different grades of HBV-related liver cirrhosis.

**Results:**

Serum endotoxin was increased with Child–Pugh (CP) class (A, B, and C). *Veillonellaceae* and *Lachnospiraceae* families were reduced in patients compared with controls. *Megamonas* and *Veillonella* genus was reduced and increased in patients compared with controls, respectively, especially in CPB and CPC groups. Correlation analysis showed that endotoxin content was significantly correlated with alcohol consumption (95% CI 0.100, 0.493), CP class (95% CI 0.289, 0.687) and *Lachnospiraceae* family level (95% CI − 0.539, − 0.122). *Firmicutes/Bacteroidetes* ratio was correlated with the level of *Lachnospiraceae* family (95% CI 0.013, 0.481), *Veillonellaceae* family (95% CI 0.284, 0.696), *Megamonas* genus (95% CI 0.101, 0.518) and *Veillonella* genus (95% CI 0.134, 0.545). All aforementioned bacteria were independent risk or protective factors for hepatitis. Alcohol consumption changed microbial community.

**Conclusions:**

Our study demonstrated that elevated *Firmicutes/Bacteroidetes* ratio, reduced *Megamonas* genus level and increased *Veillonella* genus level were indicators for HBV-related liver cirrhosis. Alcohol-related pathogenesis was associated with the changed microbial community.

## Highlights

1. Serum endotoxin increased in patients with higher Child–Pugh class.
2. Alcohol consumption increased endotoxin in Child–Pugh class A patients.
3. *Megamonas* and *Veillonella* was reduced and increased in patients vs. control, respectively.
4. Alcohol consumption changed microbial community in patients.
5. *Firmicutes/Bacteroidetes* ratio increment correlated with hepatitis pathogenesis.

## Background

Hepatitis B virus (HBV) infection is a global public health problem. The prevalence of chronic HBV infection ranges from 0.20 to 22% in different countries [1–3]. There are increasing evidence showing the relevance of intestinal microbiota with hepatosis, like HBV-related liver cirrhosis [4, 5].

Chronic HBV infection reduces liver functions and eventually gives rise to liver cirrhosis, hepatitis and hepatocellular carcinoma (HCC). The significant dissimilarities in intestinal microbial community between chronic hepatitis B or HBV-related liver cirrhosis and healthy controls have been widely reported [4, 6]. With comparison to healthy controls, patients with chronic hepatitis B showed abundant *Anaerostipes* taxon [7]. Liu et al. reported that patients with HBV-related HCC had reduced levels of pro-inflammatory bacteria like *Escherichia*–*Shigella* and increased level of anti-inflammatory bacteria like *Faecalibacterium*, while patients with non-HBV and non-hepatitis C virus (HCV) HCC showed opposite results [8]. Additionally, the altered abundance of gut microbial community has been identified in non-alcoholic fatty liver disease (NAFLD) and is deemed to be associated with progression into non-alcoholic steatohepatitis (NASH) [5, 9]. However, alcohol consumption itself induces injury and inflammation in the intestine and liver, and dissimilarity in microbial community [10, 11]. For instance, Stearns et al. showed that alcohol consumption increased the ratio of *Firmicutes* to *Bacteroidetes* [11], which is used as a parameter of obese [12, 13], lipid metabolism [14] and insulin resistance [15]. These observations showed gut microbiota dysbiosis was relevant to HBV infection and might benefit in the progression to severe liver failure like HCC and liver disease. However, the association of microbial community dissimilarity with patients with HBV-related liver cirrhosis was unclear till now.

We performed this study to investigate the microbial community dissimilarity in healthy and patients with different grades of HBV-related liver cirrhosis. The present study included a study cohort of 80 patients with HBV-related liver cirrhosis at Child–Pugh class A, B, C and 20 healthy controls without known liver diseases. The microbial community diversity in different groups were analyzed and compared. And alcohol history of each patient was asked for the analysis of alcohol consumption-related factors. The bacteria associated with hepatitis were identified. According to the reported relation of *Firmicutes*/*Bacteroidetes* ratio to diseases [15], we also performed logistics analysis to identify the correlation of this ratio with disease progression.

## Methods

### Patient population

Before experiments, an ethical approval and consent to participate (LDYYLL2018-142) was obtained from the ethical committees of First Hospital of Lanzhou University, Lanzhou, China. This study enrolled 80 patients (han Chinese) with HBV-related cirrhosis (including 55 males and 25 females, aged 42.30 ± 13.15 years) admitted in our hospital from July 2018 to Dec 2018. All patients with HBV-related cirrhosis were staged according to the Child–Pugh (CP) class A (CPA, n = 30), B (CPB, n = 31) and C (CPC, n = 19). All patients did not receive probiotics and antibiotics for 8 weeks prior to admission. Patients with solid organ transplantation, HCC, drug-induced liver injury, autoimmune liver disease and alcoholic fatty liver were excluded. In addition, patients with specific food habits, like vegetarians and lactose lovers, were excluded from our study. Twenty healthy volunteers (age- and sex-matched) without known diseases were randomly selected from the physical examination center of our hospital. Use of antibiotics within 8 weeks prior sample collection was forbidden. Alcohol history and consumption (white liquor) of all patients was asked. Fasting blood and stool samples were collected from all participants with informed consent. All samples were set at − 80 °C before examination. This study was performed according to the Declaration of Helsinki (1975).

### Blood biochemical indicator detection

The serum diamine oxidase (DAO), d-lactate, endotoxin and HBV-DNA was detected using highly sensitive human DAO ELISA kit, lactic acid and bacterial endotoxin assay kits (JY-Po-Color DLT Set; Zhongsheng jinyu diagnostic technology co. LTD., Beijing, China), respectively. Three duplicates were setup for each sample.

### DNA extraction and sample preparation

Total DNA samples were extracted from stool samples using MoBioPowersoil DNA extraction kits (MoBio, Carlsbad, CA, USA) following the manufacturer’s instructions. DNA quality was determined using 1% agarose gel electrophoresis. Amplification of the V4 region of the 16S rRNA gene was conducted using the 515 (Forward, 5′-GTGCCAGCMGCCGCGGTAA-3′)/806 (Reverse, 5′-GGACTACHVGGGTWTCTAAT-3′) primers and TransGen AP221-02: TransStartFastpfu DNA Polymerase (TransGen Biotech, Beijing, China). Amplification of 20 µL reactions was implemented on Applied Biosystems GeneAmp^®^ 9700 (Applied Biosystems, Foster City, CA, USA) at 94 °C for 4 min, followed by 30 cycles of 94 °C for 40 s, 58 °C for 30 s, and 72 °C for 45 s, and followed by a final extension at 72 °C for 10 min. Three duplicates were set up for each sample. PCR products from one sample were pooled and then gel purified (2% agarose gel) using an AxyPrep DNA Gel Extraction kit (Axygen Biosciences, Hangzhou, China). DNA quantification was performed using QuantiFluor™ (Promega, Lyon, France). DNA library construction was carried out using the TruSeq^®^ DNA PCR-Free Sample Preparation Kit (Illumina, San Diego, USA) and 16S rRNA gene sequencing was conducted on the Illumina MiSeq platform (Illumina, San Diego, USA; 2 × 250 bp PE).

### Data processing and bioinformatics analysis

Raw reads were obtained and merged using FLASH (version 1.2.7; http://ccb.jhu.edu/software/FLASH/). Low-quality bases were trimmed using the Trimmomatic program (version 0.36, http://www.sadellab.org/cms/index.php?page=trimmomatic). The sorting of microbial operational taxonomic units (OTUs) and taxonomic assignments was performed using Qiime (v1.9.1; http://qiime.org/scripts/split_libraries_fastq.html). Chimera sequences in OTUs were removed using Usearch (version 7.1, http://drive5.com/uparse/). Annotation of OTUs was performed in SILVA’s SSU rRNA database (http://www.arb-silva.de/). The OTUs’ alpha diversity estimators, including community richness (Chao1), community diversity (Shannon and Simpson indices) and sequencing depth (Good’s coverage) were analyzed using Mothur (version v.1.30.1, http://www.mothur.org/wiki/Schloss_SOP#Alpha_diversity). Rarefaction curves were analyzed using Mothur. OTUs with at 97% identity and > 1% relative abundance were retained for further analysis. Beta diversities (Bray–Curtis dissimilarity) between samples were analyzed using Qiime. Principal co-ordinates analysis (PCoA; Bray–Curtis distance) was performed using R programing language.

### Statistical analyses

All clinical data were expressed as mean ± standard deviation or median and range (Q1–Q3) and were analyzed using SPSS 22.0. Comparisons in blood biochemical indicators were performed using non-parametric Mann–Whitney U test, Kruskal–Wallis H test or χ^2^ test. Differences between alcohol and non-alcohol consumed patients were analyzed using Mann–Whitney U test. For gut microbiota analysis, non-parametric Kruskal–Wallis H test and Wilcoxon rank-sum test was used for the differences in alpha and beta diversities. Differences in taxonomies were analyzed using non-parametric Kruskal–Wallis sum-rank test and LDA Effect Size (LEfSe) software (http://huttenhower.sph.harvard.edu/galaxy/root?tool_id=lefse_upload). Differences in the relative abundances of OTUs of dominant bacteria were analyzed using Welch’s t test or Mann–Whitney U test. Spearman correlation coefficients (r) between dominant bacteria and blood biochemical indicators were identified. Logistic regression analysis was performed to identify the indicators for disease progression. p < 0.05 was considered statistically significant.

## Results

### Differences in serum indicators

There was no difference in the age, male ratio, and alcohol consumption frequency among the three groups (Table 1). The difference in the content of serum DAO (range 0.86–14.20 U/L), d-lactate (2.46–20.75 mg/L) and endotoxin (0.12–23.59 U/L) are shown in Table 1. Serum DAO and d-lactate contents were equivalent in patients with CP class A (n = 30), B (n = 31) and C (n = 19) HBV-related cirrhosis. Serum endotoxin and HBV-DNA contents were increased in patients with higher CP classes (p = 0.000 and p = 0.000, respectively; Table 1). Alcohol consumption increased the serum endotoxin in all patients (p = 0.006, Table 2); endotoxin and d-lactate contents in CPA patients (p = 0.008 and 0.017, respectively); but decreased DAO content in CPC patients (p = 0.035).

**Table 1:** Demographical characteristics and blood biochemical indicators in patients

Indicators	CPA^a^	CPB^b^	CPC^c^	p
Patients	30	31	19	
Age (years)	46.5 (34–67)	48.7 (32–67)	52.8 (38–57)	0.571*
Gender (male)	66.67% (20/30)	63.33% (19/31)	68.42% (13/19)	0.851^@^
Alcohol consumption (Yes/no)	23.33% (7/30)	48.38% (15/31)	47.37% (9/19)	0.114^@^
DAO (U/L)	2.26 (1.88–2.61)	2.37 (1.85–2.72)	2.39 (1.54–2.98)	0.799*
d-lactate (mg/L)	10.29 (8.35–12.83)	8.53 (7.20–12.78)	11.27 (6.320–14.260)	0.570*
LPS (U/L)	4.70 (2.88–8.26)	7.26 (5.72–9.66)	11.64 (9.970–17.000)	0.000*
HBV-DNA	0	0 (0–1.05 × 10^4^)	1.302 × 10^6^ (0–5.510 × 10^6^)	0.000*

*and ^@^, differences were analyzed using non-parametric Kruskal–Wallis H test and χ^2^ test, respectively

^a,b,c^Patients with Child–Pugh class A (n = 30), B (n = 31) and C (n = 19) hepatitis B or hepatitis B virus-related cirrhosis

**Table 2:** Analysis of blood biochemical indicators between patients with and without alcohol consumption

Indicators	CPA^a^		p	CPB^b^		p	CPC^c^		p	Total (median)		p
	Yes	No		Yes	No		Yes	No		Yes	No	
DAO (U/L)	2.38 (2.18–2.49)	1.94 (1.82–2.82)	0.441	2.41 (2.27–3.52)	2.24 (1.82–2.65)	0.217	1.71 (1.27–2.44)	2.83 (2.08–3.12)	*0.035*	2.38 (1.87–2.55)	2.24 (1.82–2.85)	0.825
d-lactate (mg/L)	13.24 (8.77–19.12)	10.14 (7.95–12.30)	*0.017*	8.08 (7.23–12.56)	8.68 (6.89–10.88)	0.959	9.99 (6.41–13.18)	11.85 (3.43–14.32)	1.000	9.99 (7.23–13.66)	10.04(7.41–12.72)	0.904
LPS (U/L)	8.09 (5.60–11.64)	3.74 (2.67–6.06)	*0.008*	7.38 (5.61–11.54)	6.22 (4.70–9.10)	0.413	11.99 (10.50–16.22)	11.48 (8.40–17.39)	0.780	9.52 (5.914–11.99)	6.06 (3.23–9.31)	*0.006*

^a,b,c^Patients with Child–Pugh class A (n = 30), B (n = 31) and C (n = 19) hepatitis B or hepatitis B virus-related cirrhosis. Differences were analyzed using non-parametric Mann–Whitney U test

### Sequencing depth estimation

**Fig. 1:**
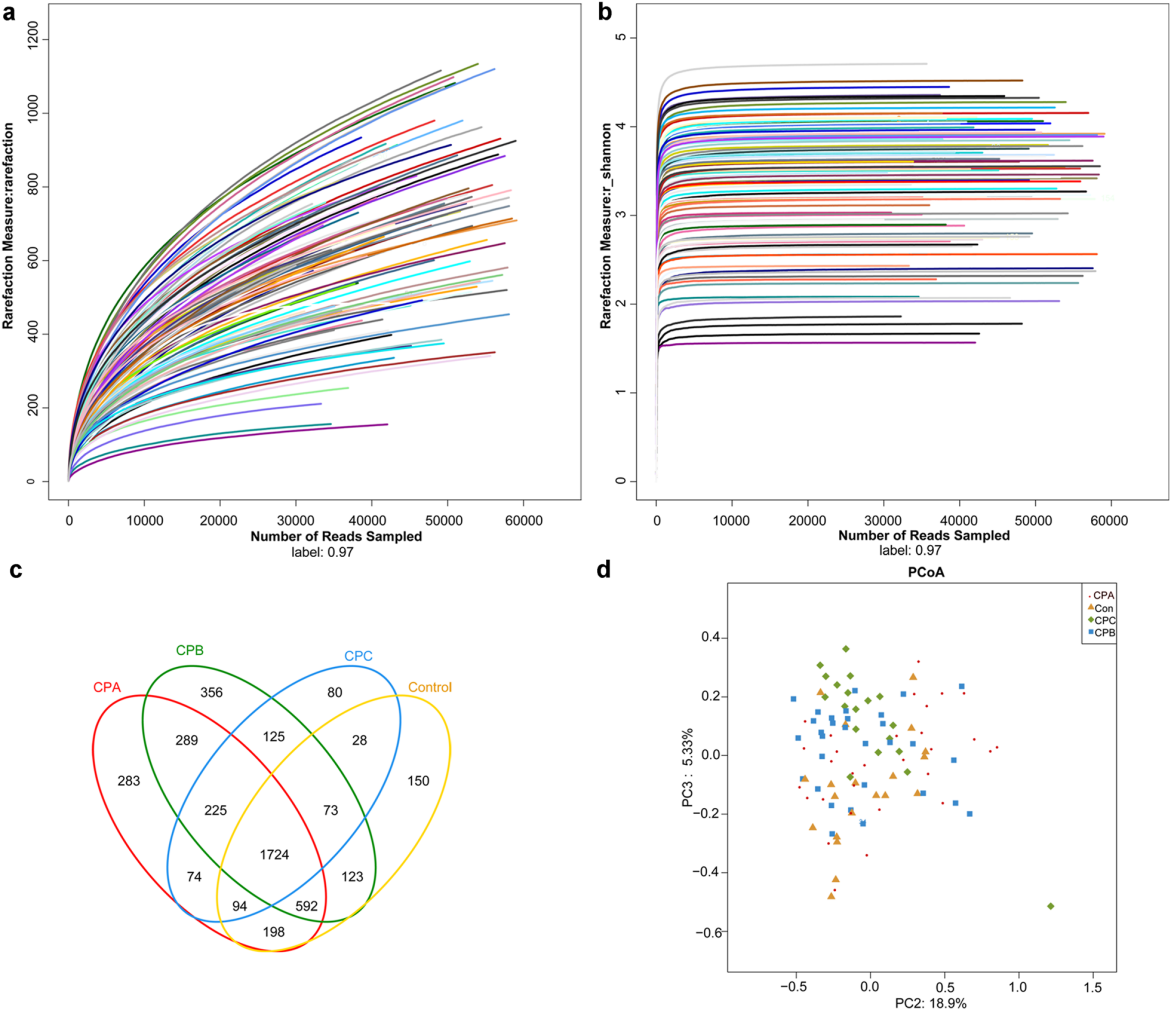
Estimation of sequencing. **a**, **b** The rarefaction curves and Shannon–Wiener curves of samples, respectively. **c** The Venn diagram of the OTUs in groups. **d** The principal co-ordinates analysis (PCoA) plot of samples

Illumina sequencing generated 4,661,253 sequences (ranging 251–300 bp), with an average length of 255.76 bp. These sequences represented 4414 OTUs with 97% identity after data processing. Rarefaction curves and Shannon–Wiener curves reached plateaus, indicating sufficient sequencing depths for microbial diversity analysis (Fig. 1a, b). Venn diagram showed there were 1724 overlapped OTUs (Fig. 1c). PCoA based on the Bray–Curtis distance showed there was no distinct clustering of species groups (Fig. 1d).

### Microbial community dissimilarity in patients

**Fig. 2:**
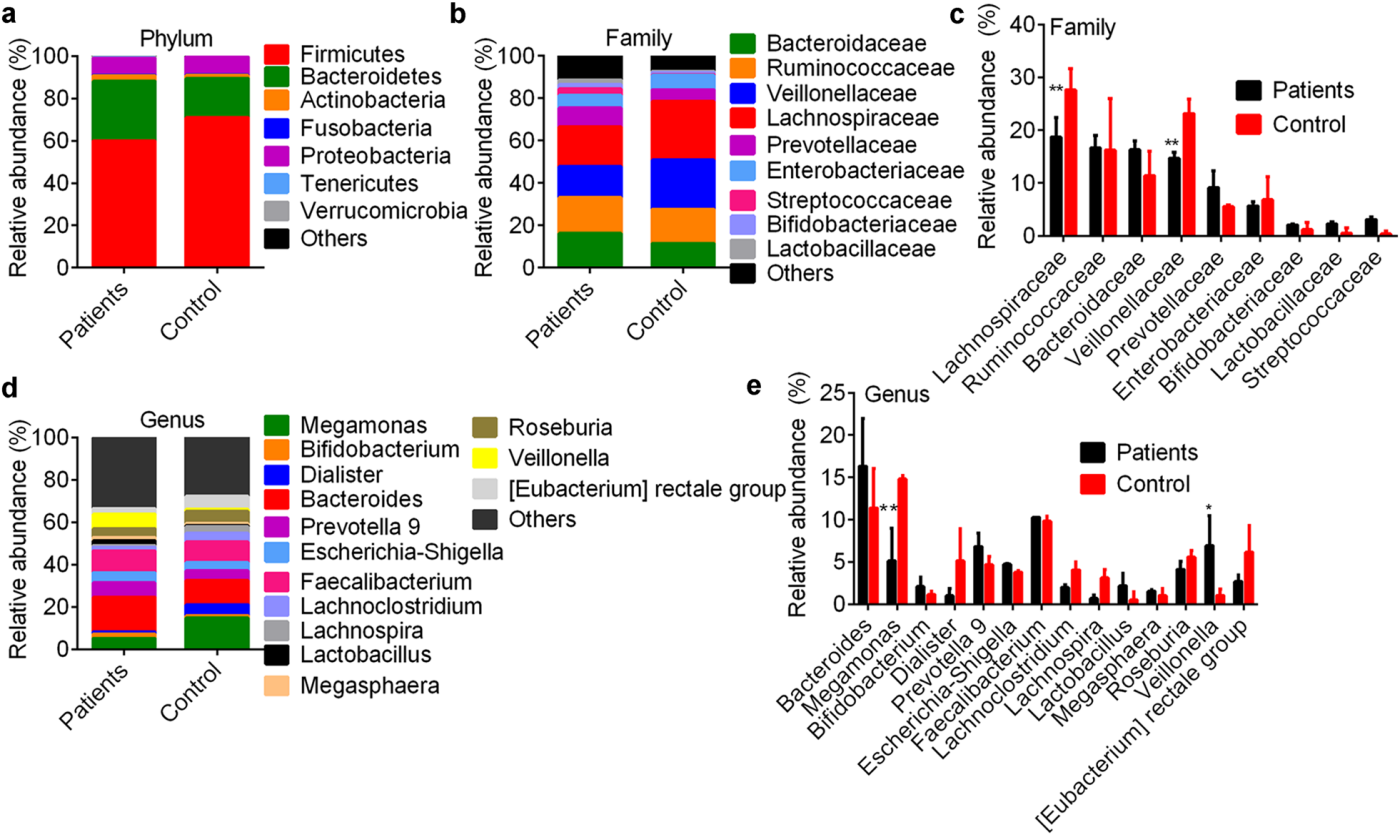
Bacterial community in health controls and patients. Dominant bacterial abundance at the phylum level (**a**), family (**b**, **c**) and genus level (**d**, **e**). ** q < 0.01 vs. control by Wilcoxon rank-sum test

**Fig. 3:**
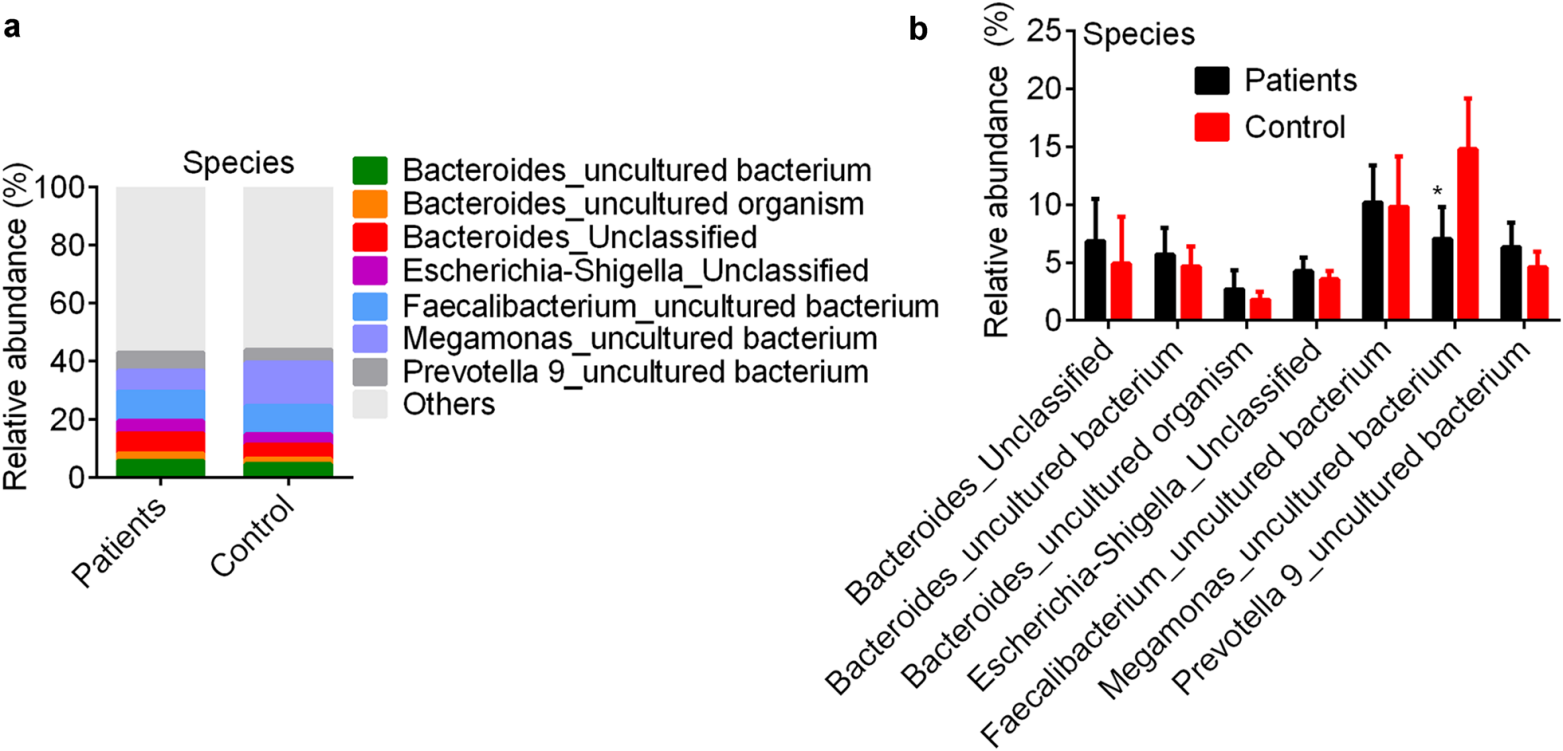
**a** showes species abundance (> 1%) between health controls and patients. **b** showes statistically significant effects of species abundance (> 1%) between groups. * p < 0.05 vs. control by Wilcoxon rank-sum test

Microbial diversity analysis showed that the relative abundances of *Firmicutes* and *Bacteroidetes* phylum in patients were equivalent with that in control (Fig. 2a). At the family level, *Lachnospiraceae* (*Firmicutes* phylum, 18.70 ± 3.20%) and *Veillonellaceae* (*Firmicutes* phylum, 14.73 ± 3.13%) relative abundances in patients were significantly lower than that in controls (27.64 ± 2.17%, p < 0.0001; and 23.16 ± 3.73%, p < 0.0001; Fig. 2b, c). At the level of genus, *Megamonas* (*Veillonellaceae* family) relative abundance in patients (5.14 ± 1.17%) was significantly lower than that in controls (14.80 ± 4.67%, p < 0.0001, Fig. 2d, e), and *Veillonella* genus showed opposed trends. Species taxonomy showed the dominant bacteria were uncultured bacteria of *Faecalibacterium*, *Megamonas* and *Bacteroide*s. Patients had low abundance of an uncultured species of *Megamonas* (7.06 ± 2.74%) compared with healthy controls (14.77 ± 3.39%, p < 0.0001, Fig. 3a, b).

### Microbial communities in patients with different CP classes

**Fig. 4:**
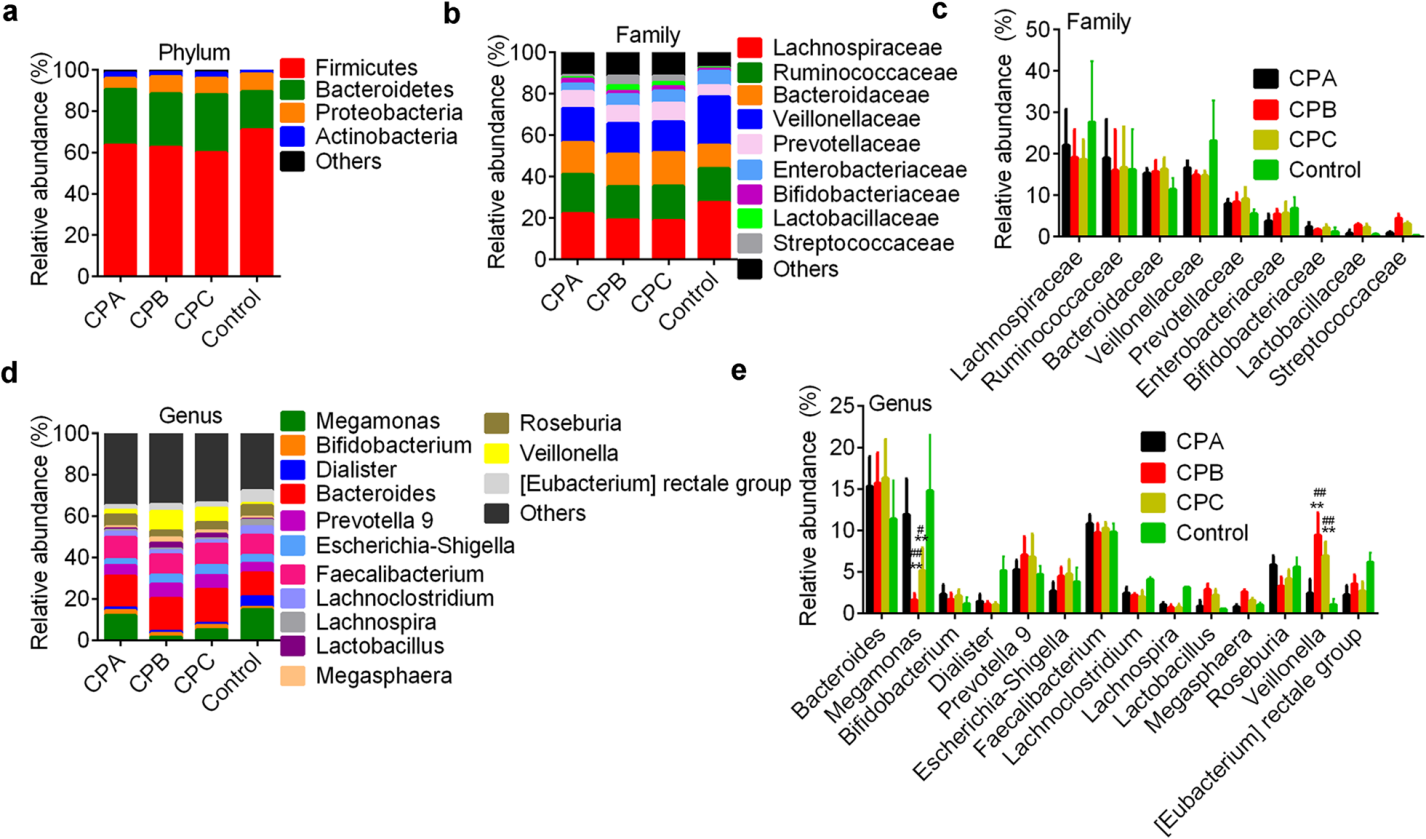
Bacterial community in health controls and patients with different classes. Dominant bacterial abundance at the phylum level (**a**), family (**b**, **c**) and genus level (**d**, **e**). ** p < 0.01 vs. control by non-parametric Kruskal–Wallis test. ^#^p < 0.05 and ^##^p < 0.01 vs. CPA (patients with Child–Pugh class A hepatosis, respectively. CPA, CPB and CPC, patients with Child–Pugh class A (n = 30), B (n = 31) and C (n = 19) hepatosis (hepatitis B or hepatitis B virus-related cirrhosis)

**Fig. 5:**
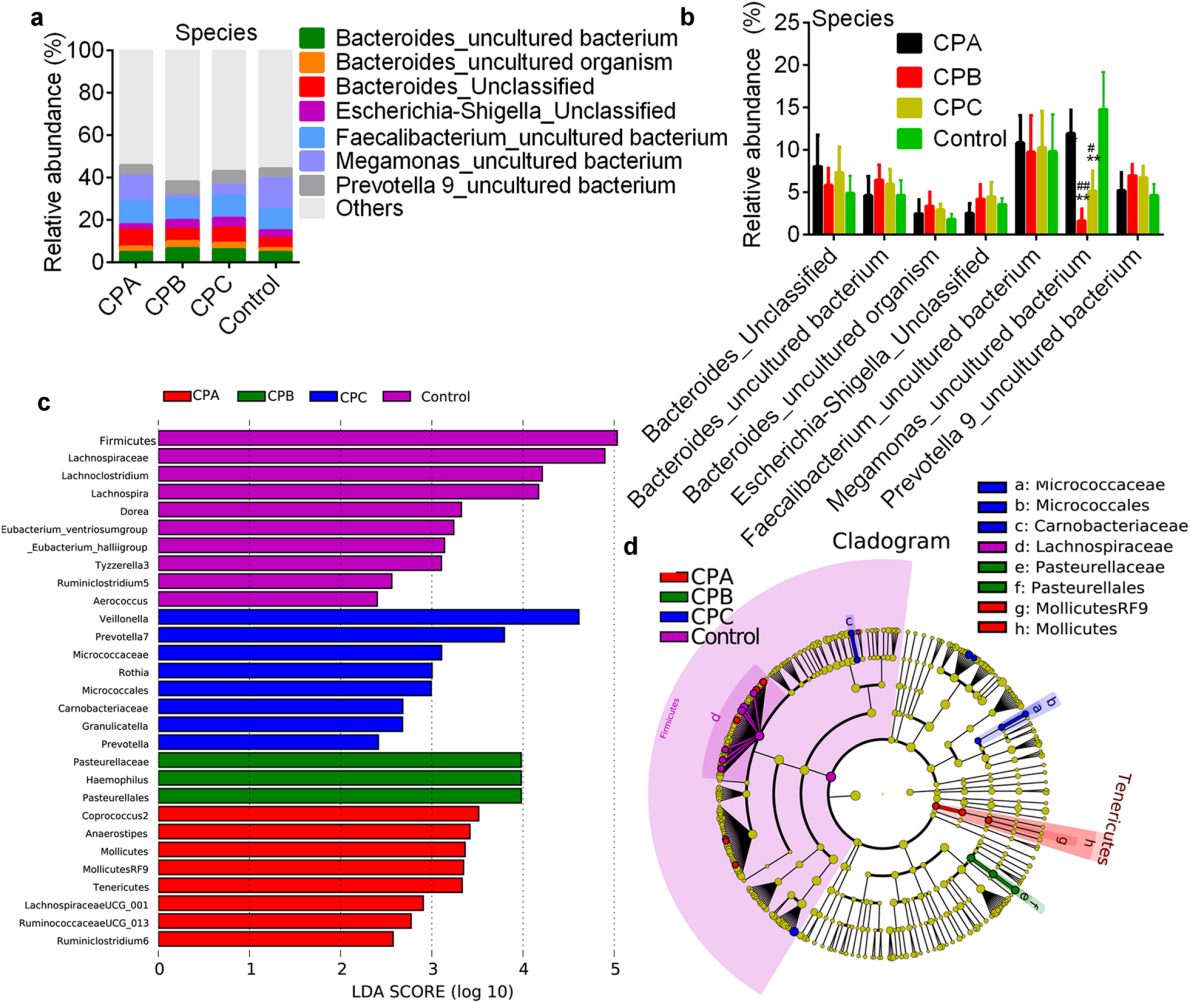
**a** showes species community (> 1% abundance) in health controls and patients with different classes.** b** showes the statistically significant effects between health controls and patients with different classes. *p < 0.05, ** p<0.01 vs. control by Wilcoxon rank-sum test. ^#^p < 0.05 and ^##^p < 0.01 vs. CPA (patients with Child–Pugh class A hepatosis, respectively. CPA, CPB and CPC, patients with Child–Pugh class A (n = 30), B (n = 31) and C (n = 19) hepatosis (hepatitis B or hepatitis B virus-related cirrhosis). **c** shows the LEfSe results in these four groups. **d** shows the cladogram results of all the bacteria

We further performed the pairwise comparison between patients with different CP classes and controls in order to investigate the microbial community dissimilarity in patients with different disease severities. Microbial community analysis showed there were no differences in the relative abundances of bacteria at the phylum (Fig. 4a) and family level (Fig. 4b, c). *Lachnospiraceae* (18.75–27.64%), *Ruminococcaceae* (*Firmicutes* phylum, 16.02–19.07%), *Veillonellaceae* (14.73–13.16%) and *Bacteroidaceae* (*Bacteroidetes* phylum, 11.39–15.70%) were dominant families in all groups. At the genus level, *Faecalibacterium* (9.71–10.82%), *Megamonas* (1.61–14.80%) and *Bacteroide*s (11.39–16.33%) were dominant taxonomies. Patients with CPB and CPC HBV-related liver cirrhosis had lower *Megamonas* level (1.61 ± 1.10% and 5.14 ± 3.11%) and higher *Veillonella* level (9.43 ± 2.18% and 6.93 ± 2.67%) compared with control (14.30 ± 5.11% and 1.05 ± 0.84%, respectively; p < 0.0001 for both) and patients with CPA disease (11.95 ± 2.31% and 2.41 ± 1.55%; p < 0.0001, Fig. 4d, e). Species taxonomy dissimilarity analysis showed uncultured bacteria of *Faecalibacterium*, *Megamonas* and *Bacteroide*s were dominant bacteria in all groups (Fig. 5a, b). The relative abundance of an uncultured species of *Megamonas* in patients with CPB (1.61 ± 0.74%) and CPC (5.13 ± 1.82%) disease was dramatically lower than that in controls (14.77 ± 3.39%, p < 0.0001) and patients with CPA disease (11.93 ± 2.92%, p < 0.0001, Fig. 5b). LEfSe analysis showed that *Mollicutes* family (*Tenericutes* phylum), *Lachnospiraceae* family, *Micrococcales* family (*Actinobacteria* phylum) and *Pasteurellales* (*Proteobacteria* phylum) were important bacteria in CPA, CPB, CPC and control groups (Fig. 5c, d).

### Microbial community dissimilarity by alcohol consumption

**Fig. 6:**
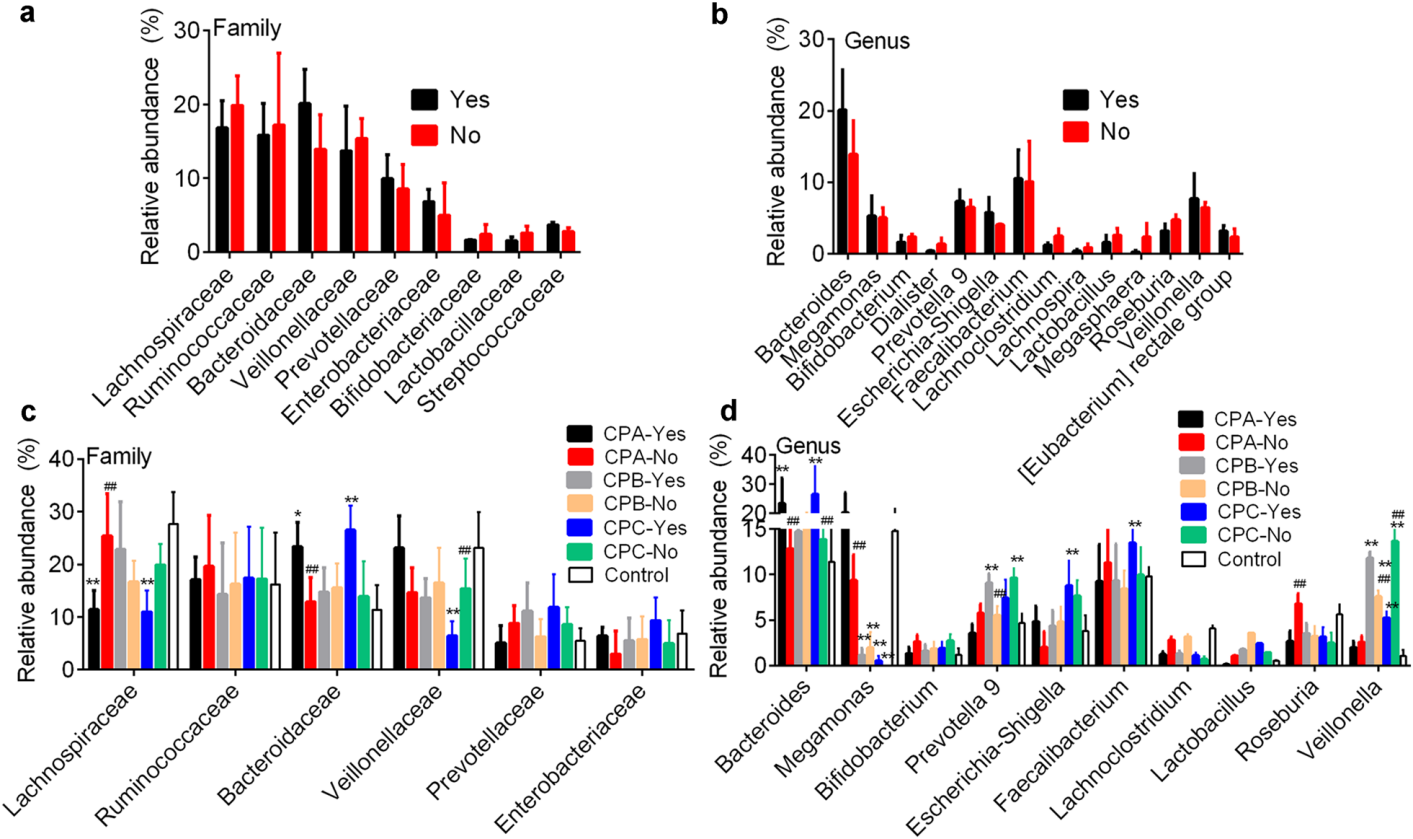
**a** and **b** show the bacteria community dissimilarity in patients with (Yes) and without (No) alcohol consumption at family and genus level, respectively. **c** and **d** show the bacteria community dissimilarity in different groups with (Yes) and without (No) alcohol consumption at family and genus level, respectively. *p < 0.05 and **p < 0.01 vs. control by Mann–Whitney test, respectively. ^#^p < 0.05 and ^##^p < 0.01 vs. patients without alcohol consumption (No), respectively. CPA, CPB and CPC, patients with Child–Pugh class A (n = 30), B (n = 31) and C (n = 19) hepatosis (hepatitis B or hepatitis B virus-related cirrhosis)

In order to investigate the influence of alcohol consumption on microbial community dissimilarity, patients were divided into two groups: with and without alcohol consumption. There was no microbial community dissimilarity in the relative abundance of dominant phyla (data not shown), families (Fig. 6a) and genera (Fig. 6b) between the two groups. In patients within the same CP class, alcohol consumption induced significant microbial community dissimilarity at the family and genus levels (Fig. 6c, d). Alcohol consumption decreased *Lachnospiraceae* family (11.41 ± 3.68% vs. 25.37 ± 8.03%, p < 0.0001) and *Veillonellaceae* family (6.43 ± 2.73% vs. 15.39 ± 5.66%, p < 0.0001) in CPA patients; and increased *Bacteriodaceae* family in CPA patients (23.37 ± 4.67% vs. 12.94 ± 5.13%, p < 0.0001; Fig. 6c). At the genus level, we found alcohol consumption increased *Bacteroide*s (CPA and CPC) and *Megamonas* (CPA) and *Veillonella* (CPB) level, respectively (Fig. 6d). These differences demonstrated that alcohol consumption caused microbial community dissimilarity in patients with HBV-related cirrhosis.

### Correlation analysis for dominant bacteria

To investigate the correlation of microbial community dissimilarity in patients with HBV-related liver cirrhosis, we performed Spearman correlation analysis. The results showed that endotoxin was correlated with alcohol consumption (r = 0.314, 95% CI 0.100, 0.493, p = 0.006) and CP class (r = 0.501, 95% CI 0.289, 0.687, p = 0.000). The relative abundance of *Lachnospiraceae* family was negatively correlated with endotoxin (r = − 0.345, 95% CI − 0.539, − 0.122, p = 0.002), HBV-DNA content (r = − 0.315, 95% CI − 0.497, − 0.119, p = 0.006), CP class (r = − 0.410, 95% CI − 0.589, − 0.202, p = 0.001) and *Bacteroidetes* phylum level (r = − 0.247, 95% CI − 0.453, 0.017, p = 0.033), while positively correlated with *Firmicutes* phylum level (r = 0.395, 95% CI 0.166, 0.588, p = 0.000) and *Firmicutes/Bacteroidetes* ratio (r = 0.278, 95% CI 0.013, 0.481, p = 0.016). *Veillonella* genus abundance was positively correlated with CP class (r = 0.396, 95% CI 0.211, 0.573, p = 0.000), HBV-DNA content (r = 0.403, 95% CI 0.207, 0.581, p = 0.000), *Veillonellaceae* family level (r = 0.554, 95% CI 0.313, 0.761, p = 0.000) and *Firmicutes/Bacteroidetes* ratio (r = 0.356, 95% CI 0.134, 0.545, p = 0.000; Table 3). *Veillonella* genus was negatively correlated with *Bacteroidetes* phylum level (r = − 0.374, 95% CI − 0.564, 0.157, p = 0.001). The *Firmicutes/Bacteroidetes* ratio was positively correlated with *Lachnospiraceae* family level (r = 0.278, 95% CI 0.013, 0.481, p = 0.016), *Veillonellaceae* family level (r = 0.509, 95% CI 0.284, 0.696, p = 0.000), *Megamonas* genus level (r = 0.321, 95% CI 0.101, 0.518, p = 0.005), *Veillonella* genus level (r = 0.356, 95% CI 0.134, 0.545, p = 0.002), and *Firmicutes* phylum level (r = 0.876, 95% CI 0.757, 0.953, p = 0.000), while *Bacteroidetes* phylum (r = − 0.985, 95% CI − 0.995, − 0.961, p = 0.000) was negatively correlated with these bacteria (Table 3). This was true for *Firmicutes* phylum.

**Table 3:** Spearman correlation coefficients between intestinal microbial community dissimilarity and baseline characters indicators in patients

Indicators												
d-lactate	1.000											
LPS	0.112	1.000										
Alcohol consumption	0.066	0.314**	1.000									
CP class	− 0.063	0.501**	0.224	1.000								
DAO	− 0.063	0.154	0.007	0.088	1.000							
*Lachnospiraceae* family	− 0.069	− 0.345**	− 0.175	− 0.410**	0.083	1.000						
*Veillonellaceae* family	0.138	0.038	0.016	− 0.024	0.020	− 0.145	1.000					
*Megamonas* genus	0.096	− 0.059	0.038	− 0.259*	− 0.073	− 0.202	0.550**	1.000				
*Veillonella* genus	0.066	0.225	0.149	0.396*	0.026	− 0.052	0.554**	0.001	1.000			
*Firmicutes* phylum	− 0.030	− 0.111	− 0.207	− 0.220	0.020	0.395**	0.411**	0.293*	0.199	1.000		
*Bacteroidetes* phylum	0.004	0.119	0.161	0.134	0.041	− 0.247*	− 0.522**	− 0.317**	− 0.374**	− 0.824**	1.000	
*Firmicutes/Bacteroidetes* ratio	0.004	− 0.127	− 0.163	− 0.156	0.002	0.278*	0.509**	0.321**	0.356**	0.876**	− 0.985*	1.000

* and ** notes p < 0.05 and p < 0.01 in two-tailed test

Logistics analysis showed that the abundance of *Megamonas* genus (β = 8.352 95% CI 1.863–9.467 × 10^6^, p = 0.034), *Veillonella* genus (β = 32.450, 95% CI 2.298 × 10^4^, 6.673 × 10^23^, p = 0.005) and *Firmicutes/Bacteroidetes* ratio (β = 0.011, 95% CI 1.002, 1.020, p = 0.015) were an independent risk factors of hepatitis B development; while that of *Lachnospiraceae* family (β = -8.518, 95% CI 0.000, 0.406, p = 0.028), *Veillonellaceae* family (β = − 15.311, 95% CI 0.000, 0.009, p = 0.005) were protective effect of this disease (Table 4). In patient with hepatitis B, none of the aforementioned bacteria and blood biochemical diversities was significantly influenced by alcohol consumption (Table 5). Alcohol consumption showed borderline significance in elevating *Megamonas* genus level (p = 0.055, β = 35.693, 0.476–2.129 × 10^31^, p = 0.055). These results showed that alcohol consumption was not the independent risk factor for serum d-lactate, endotoxin, DAO and the microbial community dissimilarity in patients with HBV-related liver cirrhosis.

**Table 4:** The logistics analysis for the significantly changed bacteria associated with hepatitis

Indicators	β	95% CI	p
*Lachnospiraceae* family	− 8.518	0.000–0.406	0.028
*Veillonellaceae* family	− 15.311	0.000–0.009	0.005
*Megamonas* genus	8.352	1.863–9.467 × 10^6^	0.034
*Veillonella* genus	32.450	2.298 × 10^4^ to 6.673 × 10^23^	0.005
*Firmicutes* phylum	7.507	0.804–4.119 × 10^6^	0.057
*Bacteroidetes* phylum	5.919	1.538–2.575 × 10^5^	0.076
*Firmicutes/Bacteroidetes* ratio	0.011	1.002–1.020	0.015

**Table 5:** The logistics analysis for the indicators associated with alcohol consumption in patients

Indicators	β	95% CI	p
*Lachnospiraceae* family	2.611	0.030–6.082 × 10^3^	0.402
*Veillonellaceae* family	− 32.015	0.000–10.098	0.079
*Megamonas* genus	35.693	0.476–2.129 × 10^31^	0.055
*Veillonella* genus	36.621	0.135–4.753 × 10^32^	0.063
*Firmicutes* phylum	− 2.917	0.000–20.298	0.335
*Bacteroidetes* phylum	0.376	0.013–163.775	0.876
*Firmicutes/Bacteroidetes* ratio	− 0.003	0.986–1.007	0.522
d-lactate	0.059	0.931–1.209	0.376
LPS	0.113	0.971–1.292	0.119
CP classes	0.161	0.475–2.905	0.728
DAO	− 0.066	0.594–1.477	0.778

## Discussion

Our present study demonstrated that intestinal microbial community diversity in patients with HBV-related liver cirrhosis was different from that in healthy controls. The intestinal microbial community dissimilarity, like reduced *Megamonas* genus (*Veillonellaceae* family) level, *Lachnospiraceae* family level as well as increased *Veillonella* genus (*Veillonellaceae* family) level, was associated with CP classes in patients. In addition, we confirmed that the *Firmicutes/Bacteroidetes* ratio was positively correlated with the levels of above-mentioned bacteria. Additionally, alcohol consumption did not obviously altered the levels of these bacteria and serum parameters including d-lactate, endotoxin and DAO contents in patients with HBV-related liver cirrhosis.

The *Firmicutes/Bacteroidetes* ratio was reported to be associated with the morbidity and pathogenesis of several chronic diseases like obesity or diabetes [13–15]. Obese population, both children and adults, had elevated *Firmicutes/Bacteroidetes* ratio compared with controls with normal weight [13, 16]. On contrast, decreased *Firmicutes/Bacteroidetes* ratio in obese individuals was identified to be related with weight loss [17]. Cui et al. showed that the elevated the *Firmicutes/Bacteroidetes* ratio in pigs was correlated with reduced fatty acid synthase (*FASN*) and acetyl-CoA carboxylase α (*ACCα*) mRNA levels in liver, but opposed trends in subcutaneous fat. The inhibition of *ACCα* suppresses *FASN* and thus inhibits the synthesis of fatty acid from free fatty acids (FFAs) like saturated straight-chain FAs [18]. Decreased *FASN* level was detected in adipose tissue from hypertensive individuals and subcutaneous adipose tissue from obese subjects [18–20]. What’s more, *FASN* has a negative correlation with insulin resistance [21]. Consistently, our present study suggested that the increased *Firmicutes/Bacteroidetes* ratio was an independent risk facor for HBV-related liver cirrhosis, indicating the alterations in lipid metabolism and FAs biosynthesis in patients.

*Megamonas* and *Veillonella* genus were identified to be significantly contributed to the pathogenesis of liver disease. Both *Megamonas* and *Veillonella* genera are poorly characterized members of *Veillonellaceae* family, *Firmicutes* phylum. *Veillonella* genus is anaerobic genus [22]. However, both *Megamonas* and *Veillonella* are frequently elevated in cystic fibrosis cases [23], individuals with type I diabetes [22] and patients with primary sclerosing cholangitis with or without inflammatory bowel disease (IBD) compared with healthy controls [24, 25]. *Veillonella* genus is a producer of butyrate and short-chain fatty acids (SCFAs) [26] which contributes to anti-inflammatory response in the host [27]. The elevated and decreased level of *Megamonas* was identified in the host with IBD [24] and Behcet’s disease [28], respectively. Chakravarthy et al. detected the reduced *Veillonella dispar* level in patients with uveitis, an inflammatory disease of the eye [29]. By contrast, Matera et al. reported the increased level of *Veillonella* in the mouth flora in individuals with periodontal disease [30]. Additional in vitro evidence suggested that *Veillonella* LPS stimulated the release of cytokines like interleukin-6 (IL-6), IL-1β, IL-10 and tumor necrosis factor alpha (TNF-α) in human peripheral blood mononuclear cells [30]. These results suggested that intestinal *Veillonella* metabolites like LPS and *Veillonella*-modulated metabolites like SCFAs affect the liver pathology and inflammation in the host [9]. We found the level of *Veillonella* and *Megamonas* was positively and negatively correlated with Child–Pugh classes in patient with HBV-related liver cirrhosis, respectively. The elevated *Veillonella* genus and decreased *Megamonas* genus might suggest the unbalanced inflammatory status in patients with HBV-related liver cirrhosis compared with control.

Several observations suggested that alcohol consumption itself not only induces injury and inflammation in the intestine and liver but also causes dissimilarity in microbial community [10, 11]. Our present study confirmed that there was no dissimilarity in microbial community between patients with and without alcohol consumption. But differences were observed in microbial community between healthy controls and patients with or without alcohol consumption. This suggested the influence of alcohol consumption on the pathogenesis of disease.

Alcohol consumption increases intestinal permeability and therefore induces endotoxaemia [31, 32]. Some researchers showed the insignificantly influence of alcohol consumption on intestinal permeability and endotoxin [33]. Our correlation analysis showed that alcohol consumption was positively correlated with increased endotoxin level. Alcohol consumption upregulated endotoxin and d-lactate levels in patients with Child–Pugh class A disease, and DAO in patients with Child–Pugh class C disease. However, alcohol consumption showed non-benefit to endotoxin in patients with Child–Pugh class B and C diseases. Further regression analysis showed alcohol consumption was not an risk factor for endotoxin and aforementioned intestinal bacteria in patients with HBV-related liver cirrhosis. The increased endotoxin, d-lactate and DAO in patients with different Child–Pugh classes by alcohol consumption might due to the increased intestinal permeability. These data might reveal that alcohol consumption played an important inducer role in the pathogenesis of but a faint function the progression of HBV-related liver cirrhosis.

## Conclusions

Our present study demonstrated that the intestinal microbial community dissimilarity in patients with HBV-related liver cirrhosis compared with healthy controls. Elevated *Firmicutes/Bacteroidetes* ratio, reduced *Megamonas* genus (*Veillonellaceae* family) level, increased *Veillonella* genus (*Veillonellaceae* family) level was associated with Child–Pugh classes in patients with HBV-related liver cirrhosis. We additionally identified that alcohol consumption play important roles in the pathogenesis of HBV-related liver cirrhosis.

## Supplementary information

**Additional file 1: Figure S1.** The relative abundance of communities at phylum level. CPA, CPB and CPC, patients with Child–Pugh class A (n = 30), B (n = 31) and C (n = 19) hepatosis (hepatitis B or hepatitis B virus-related cirrhosis).

## Data Availability

The datasets used and/or analysed during the current study are available from the corresponding author on reasonable request.

## Abbreviations

HBV: chronic hepatitis B virus
HCC: hepatocellular carcinoma
HCV: non-hepatitis C virus
NAFLD: non-alcoholic fatty liver disease
NASH: non-alcoholic steatohepatitis
CP: Child–Pugh
CPA: CP class A
CPB: CP class B
CPC: CP class C
DAO: diamine oxidase
OTUs: operational taxonomic units
FASN: fatty acid synthase
ACCα: acetyl-CoA carboxylase α
IBD: inflammatory bowel disease
TNF-α: tumor necrosis factor alpha
