# Supplementary figures and images for: The intestinal microbial community dissimilarity in hepatitis B virus-related liver cirrhosis patients with and without at alcohol consumption
Source: Gut Pathog. 2019 Nov 26;11:58. doi: 10.1186/s13099-019-0337-2 (PMC6878713; doi:10.1186/s13099-019-0337-2)

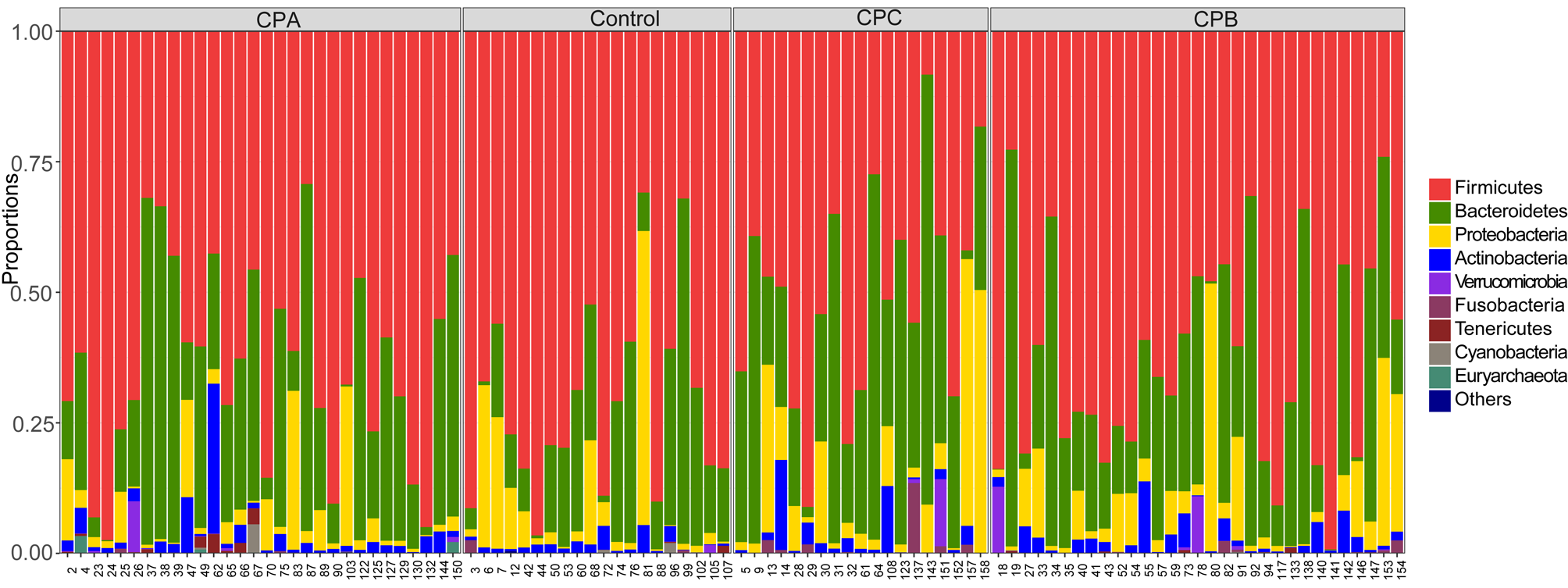

Supplement: Supplementary file 1 — Additional file 1: Figure S1. The relative abundance of communities at phylum level. CPA, CPB and CPC, patients with Child–Pugh class A (n = 30), B (n = 31) and C (n = 19) hepatosis (hepatitis B or hepatitis B virus-related cirrhosis). [file 13099_2019_337_MOESM1_ESM.tif]
